# A *Wolbachia* triple-strain infection generates self-incompatibility in *Aedes albopictus* and transmission instability in *Aedes aegypti*

**DOI:** 10.1186/s13071-018-2870-0

**Published:** 2018-05-11

**Authors:** Thomas H. Ant, Steven P. Sinkins

**Affiliations:** 1 Centre for Virus Research, University of Glasgow, Glasgow, UK; 2 Biomedical and Life Sciences, Lancaster University, Lancaster, UK; 3 Present Address: Department of Disease Control, London School of Hygiene and Tropical Medicine, Keppel Street, London, UK

**Keywords:** *Wolbachia*, *Aedes albopictus*, *Aedes aegypti*, Superinfection, Cytoplasmic-incompatibility, Population-replacement

## Abstract

**Background:**

Artificially-introduced transinfections of the intracellular bacterium *Wolbachia pipientis* have the potential to reduce the vectorial capacity of mosquito populations for viruses such as dengue and chikungunya. *Aedes albopictus* has two native strains of *Wolbachia*, but their replacement with the non-native *w*Mel strain blocks transmission of both viruses. The pattern of cytoplasmic incompatiiblity generated by *w*Mel with wild-types is bidirectional. Novel-plus-native-strain co-infection is predicted to lead to a more efficient population spread capacity; from a bi-directional to a uni-directional cytoplasmic incompatibility (CI) model.

**Results:**

A novel-plus-native-strain triple-infection in *Ae. albopictus* (*w*AlbA*w*AlbB*w*Mel) was generated. Although triple-infected females were fully reproductively viable with uninfected males, they displayed self-incompatibility. qPCR of specific strains in dissected tissues suggested that this may be due to the displacement of one of the native strains (*w*AlbA) from the ovaries of triple-infected females. When the triple strain infection was transferred into *Aedes aegypti* it displayed an unexpectedly low level of transmission fidelity of the three strains in this species.

**Conclusions:**

These results suggest that combining *Wolbachia* strains can lead to co-infection interactions that can affect outcomes of CI and maternal transmission.

**Electronic supplementary material:**

The online version of this article (10.1186/s13071-018-2870-0) contains supplementary material, which is available to authorized users.

## Background

*Wolbachia pipientis* is a maternally transmitted bacterial endosymbiont that is naturally carried by a broad range of terrestrial arthropods. The generation of novel *Wolbachia* transinfections in mosquitoes can result in reduced host permissivity for a range of pathogens [1–10]; for example, *Aedes albopictus* females transinfected with the *Wolbachia* strain *w*Mel do not transmit dengue [7] or chikungunya [8] viruses in laboratory challenges*. Wolbachia* can invade host populations by increasing the relative fitness of infected females through a pattern of crossing sterility known as cytoplasmic incompatibility (CI) [11]. CI occurs when a male infected with a *Wolbachia* strain mates with either an uninfected female or a female carrying a different non-compatible *Wolbachia* strain, and results in fertilization but inviability of the developing embryo. CI can be categorised according to whether populations show uni- or bi-directional sterility. In the simplest form of unidirectional CI, *Wolbachia*-carrying females are fully compatible with both *Wolbachia*-infected and *Wolbachia*-naïve males, but naïve females are compatible with naïve males only. *Wolbachia*-carrying females therefore do not suffer the fitness costs resulting from incompatible matings.

The fitness advantage of CI for *Wolbachia*-carriers increases with population infection frequency (with uninfected females experiencing higher proportions of incompatibility), although fitness benefits may still be appreciable at low frequencies. In the case of bidirectional CI, two interbreeding populations carry reciprocally incompatible *Wolbachia* infections; males and females of each infection type only producing viable progeny with carriers of the same infection. Females infected with the lower frequency strain will experience a higher proportion of incompatibility and will tend towards lower relative fitness. Spread through bidirectional CI is therefore typically associated with higher invasion threshold frequencies compared to unidirectional CI.

Crosses involving hosts carrying *Wolbachia* superinfections have shown that the effects of separate CI modifications can be additive [12–14]. Unidirectional CI can be produced when a superinfected line is crossed with a population carrying constituent strain(s) of the superinfection. Achieving population replacement with *Wolbachia* necessitates the release of infected female carriers; for public acceptance, logistical and economic reasons, it is desirable to release as few *Wolbachia-*carrying mosquitoes as possible to achieve self-sustaining spread. As minimum threshold frequencies for successful population invasion are typically lower under a uni-directional CI model, the generation of superinfections in naturally infected species consisting of a novel *Wolbachia* transinfection in addition to any native strains should allow for population replacement with fewer releases. Moreover, superinfected strains may allow for higher intracellular densities to be achieved than with a single constituent strain, resulting in improved pathogen inhibition. Joubert and colleagues [15] reported the generation of a superinfection in *Aedes aegypti* in which the *w*Mel and *w*AlbB *Wolbachia* strains were combined, and found more robust dengue virus inhibition in the superinfected strain compared to infections with the single constituent strains. The *w*Mel*w*AlbB line also showed a strong uni-directional CI crossing pattern with both *w*Mel and *w*AlbB-only carriers, suggesting that the *w*Mel*w*AlbB superinfection would be able to drive through single-infected populations, and could potentially rescue a control programme in the event that the dengue-blocking effectiveness of a particular strain diminished over time. Superinfections also allow the potential mixing of desirable strain phenotypes; a *w*Au*w*AlbB double-infection in *Ae. aegypti* combined the robust pathogen blocking capacity of *Wolbachia* strain *w*Au with the strong CI induction of *w*AlbB [10].

We attempted to create a native plus novel strain triple superinfection in *Aedes albopictus*, a highly invasive dengue, chikungunya and Zika virus vector with a largely suburban/semi-rural distribution. *Aedes albopictus* is naturally superinfected at high prevalence with two *Wolbachia* strains termed *w*AlbA and *w*AlbB, with *w*AlbA existing at approximately 10% the intracellular density of *w*AlbB [16]. *Aedes albopictus* was previously cured of its native *Wolbachia* infections [17] and transinfected with *w*Mel from *Drosophila melanogaster*; this line showed strong reductions in the transmission of dengue [7] and chikungunya [8] viruses. The *w*Mel infection also showed bidirectional CI with wild-type (*w*AlbA*w*AlbB) infected mosquitoes [7]. Here we report the generation of a *w*Mel*w*AlbA*w*AlbB triple superinfection in *Ae. albopictus* and assess its stability, crossing types and intracellular densities. We also describe the transfer of the triple superinfection to *Ae. aegypti* by embryonic microinjection, and assess its stability in this species.

## Results

### Superinfection generation and maternal transmission in *Ae. albopictus*

A triple strain superinfection comprising the native strains *w*AlbA, *w*AlbB and a novel strain *w*Mel was generated in *Ae. albopictus* (hereon, *w*AlbA*w*AlbB*w*Mel) through the transfer of cytoplasm from a *w*Mel infected *Ae. albopictus* line into wild-type (*w*AlbA*w*AlbB-carrying) embryos. *Aedes albopictus w*AlbA*w*AlbB*w*Mel females were out-crossed to *Wolbachia*-negative males for three generations. At G_4_ maternal inheritance rates were assessed by crossing females of the triple*-*infected line with *Wolbachia*-negative males, and the resulting progeny were assessed for *Wolbachia* infection status by strain-specific PCR. The *w*AlbA*w*AlbB*w*Mel line showed high rates of maternal transmission of all three *Wolbachia* strains to progeny: of 100 individuals tested, 97 contained all three strains while 3 carried *w*Mel only.

### CI crossing types in *Ae. albopictus*

In order to determine patterns of CI, crosses were set-up between triple-infected, wild-type and *Wolbachia*-negative mosquitoes. Crosses involving males of the *w*AlbA*w*AlbB*w*Mel line resulted in fully penetrant CI with wild-type and *Wolbachia*-negative *Ae. albopictus* females, with no eggs hatching from either cross (Table 1). When females of the *w*AlbA*w*AlbB*w*Mel infected line were crossed to *w*AlbA*w*AlbB*w*Mel males, the embryos also showed very low hatch rates, in other words the line displayed self-incompatibility. Females of the *w*AlbA*w*AlbB*w*Mel line were able to produce viable eggs when mated to *Wolbachia*-negative males and could successfully rescue (effectively overcome) the CI modification of *w*Mel-only males, but showed reduced hatch rates when crosses to wild-type males. This suggests that either (or both) of the *w*AlbA or *w*AlbB sperm modifications were causing CI in the progeny of triple-infected females.

**Table 1:** Percentage hatch rates (± SD) of eggs resulting from crosses between *Ae. albopictus* lines

		♂			
		*w*AlbA*w*AlbB	*w*AlbA*w*AlbB*w*Mel	*w*Mel	-ve
♀	*w*AlbA*w*AlbB	92.1 ± 4.2	0	0	88.2 ± 3.2
	*w*AlbA*w*AlbB*w*Mel	7.0 ± 6.7	5.8 ± 4.6	67.5 ± 13.8	65.9 ± 6.9
	*w*Mel	0	0	73.7 ± 5.6	77.9 ± 12.1
	-ve	0	0	0	88.5 ± 4.0

Each percentage shows the mean hatch rates from eggs resulting from three separate cages, each containing ten females and twenty males. For each cage > 420 eggs were assessed

### *Wolbachia* densities in *Ae. albopictus w*Alb*Aw*Alb*Bw*Mel

**Fig. 1:**
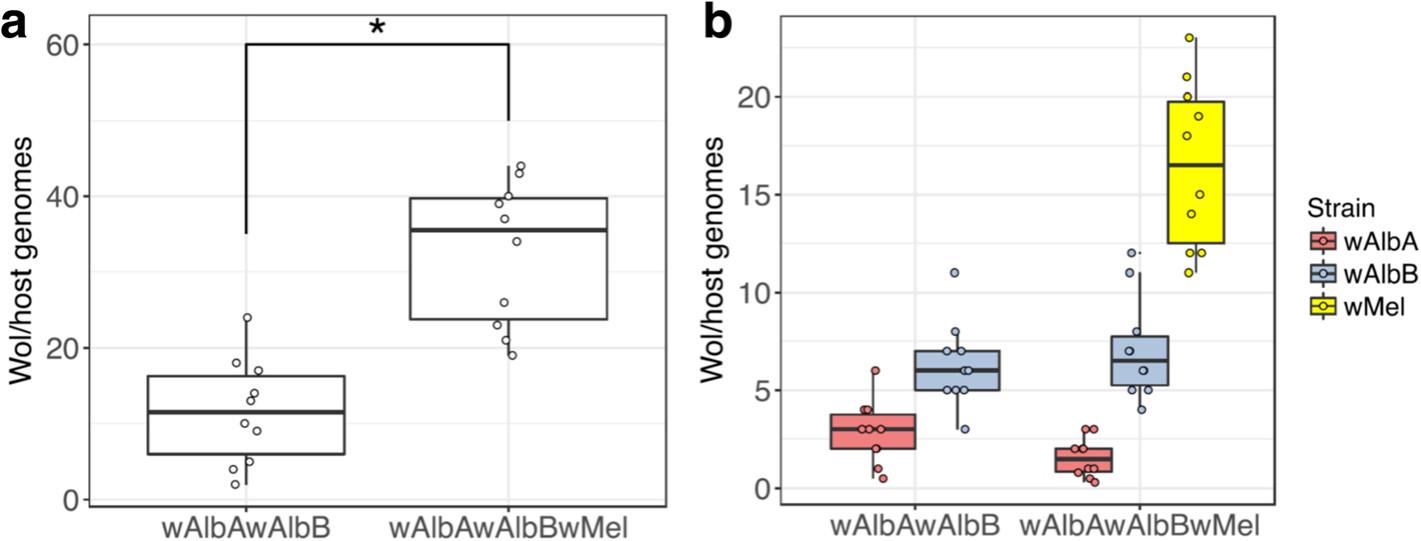
Densities of *Wolbachia* in adult *Ae. albopictus* females, measured by qPCR. Total *Wolbachia* densities (**a**) and strain-specific densities (**b**) in whole adult females. The centre of a box plot shows median *Wolbachia* density, edges show upper and lower quartiles, and whiskers indicate upper and lower extremes. Dots show values from individual biological replicates

The overall *Wolbachia* densities, and the densities of the individual *Wolbachia* strains were assessed in whole adult females and in dissected ovary, midgut and salivary gland tissues by qPCR. The overall adult female *Wolbachia* densities in the triple-infected *w*AlbA*w*AlbB*w*Mel line were found to be significantly higher than in the wild-type (one-way ANOVA, *F*_(1, 19)_ = 31.8, *P* = 0.00024) (Fig. 1a), consistent with previous findings of the *w*Mel-only infection reaching high densities in *Ae. albopictus* [7]. In whole females, the density of the *w*AlbB infection appeared to be unaffected by the presence of *w*Mel, while *w*AlbA showed a non-significant reduction (one-way ANOVA, *F*_(1, 19)_ = 3.8, *P* = 0.07) (Fig. 1b).

**Fig. 2:**
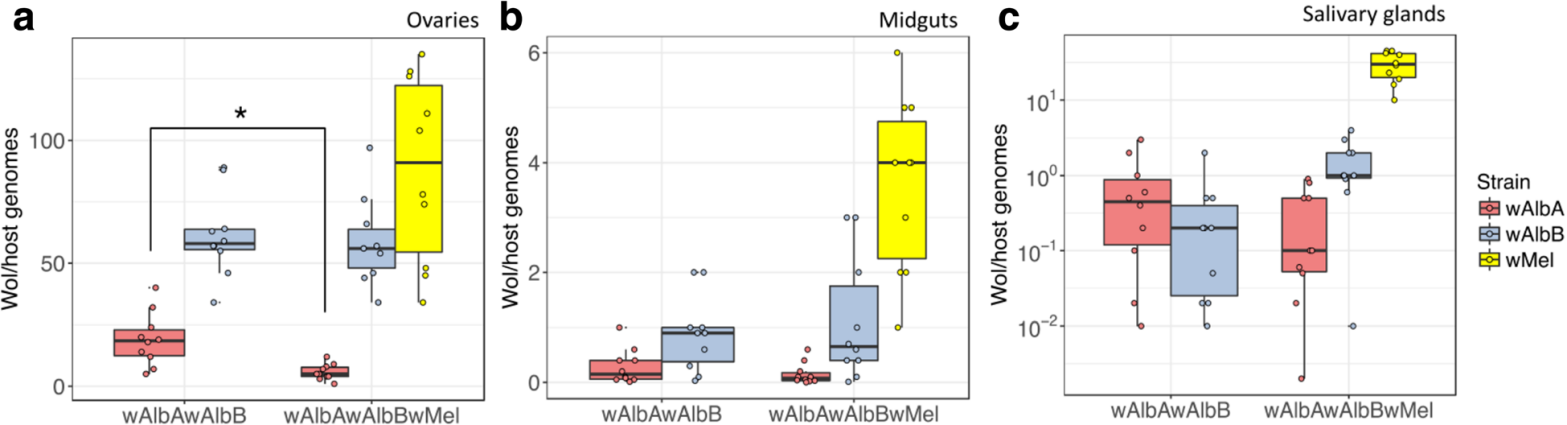
Strain-specific *Wolbachia* densities in dissected tissues. Panels show densities in ovaries (**a**), midguts (**b**) and salivary glands (**c**). Each box represents 10 biological replicates, with pools of 5 females or the tissues from 5 females per replicate. The centre of a box plot shows median *Wolbachia* density, edges show upper and lower quartiles, and whiskers indicate upper and lower extremes. Dots show values from individual biological replicates

In ovary tissues, no reduction was found in the density of *w*AlbB in the superinfected line compared to wild-type, while the density of *w*AlbA was significantly reduced (from 19.1 ± 10.8 to 5.8 ± 3.2 *Wolbachia*/host genome copies, mean ± SD; one-way ANOVA, *F*_(1, 19)_ = 13.51, *P* = 0.0017) (Fig. 2a). The reduced *w*AlbA ovary density is a potential cause of the observed self-incompatibility - with density remaining high enough for maternal transmission to occur, but insufficient in embryos to allow full rescue of the *w*AlbA CI sperm modification.

As the fitness of host and *Wolbachia* depends on maximising mosquito reproductive output, it is expected that co-evolutionary pressures will favour native *Wolbachia* localisation to the germline, and restrict densities in tissues not directly involved in vertical transmission (where high densities can lead to reductions in host fitness). Consistent with this, the native *w*AlbA and *w*AlbB strains were found to be localised largely in the ovaries (Fig. 2a), while the novel *w*Mel infection showed a far more diffuse tissue distribution, reaching relatively high densities in the somatic tissues of the midgut (Fig. 2b) and salivary glands (Fig. 2c).

### Triple-infection transfer and maternal transmission in *Ae. aegypti*

Cytoplasm from embryos of the triple-infected *Ae. albopictus* line was transferred to embryos of wild-type *Ae. aegypti* by microinjection. G_0_ females were out-crossed to wild-type males, blood-fed and individualised for oviposition. A single G_0_ female produced G_1_ progeny that were PCR-positive for all three *Wolbachia* strains (here-on *Ae.aeg-w*Mel*w*AlbA*w*AlbB). The female progeny of this line were out-crossed to wild-type males for three consecutive generations. Screening of progeny during this period revealed an unexpectedly high instability in maternal transmission of the triple infection, with progeny containing various combinations of strains recovered at high rates. At G_4_, females backcrossed to wild-type males were blood-fed and were individualised for oviposition. Individualised females were assessed for infection by PCR, and the eggs of confirmed triple-infected females were selected (20 in total). Eggs were hatched and a sample of 10 L4 larval progeny from each female were assessed for *Wolbachia* infection status by strain-specific PCR (200 larvae in total). Individuals carrying *w*AlbB-only, *w*Mel-only, and superinfections of *w*Mel*w*AlbB, *w*Mel*w*AlbA, *w*AlbA*w*AlbB and *w*Mel*w*AlbA*w*AlbB were recovered at varying rates (Table 2).

**Table 2:** Maternal transmission fidelity of *Wolbachia* strains in *Ae.aeg*-*w*AlbA*w*AlbB*w*Mel

*Wolbachia* strain(s)	% (Frequency)
*w*AlbB	11 (22/200)
*w*Mel	21 (42/200)
*w*AlbB*w*Mel	2 (4/200)
*w*AlbA*w*Mel	6.5 (13/200)
*w*AlbA*w*AlbB	14 (28/200)
*w*AlbA*w*AlbB*w*Mel	45.5 (91/200)

Eggs from crosses between triple-infected females and wild-type males were hatched and a proportion of randomly selected L4 larvae were screened for infection status by *Wolbachia* strain-specific PCR. Numbers show percentage frequency and parenthesis provide actual numbers

The *Ae. aegypti w*AlbA*w*AlbB line was out-crossed to wild-type males and displayed complete (100%) maternal transmission of both strains over a large number of generations (screening > G10), suggesting that the presence of *w*Mel was the cause of the observed transmission infidelity in the triple-strain superinfected line.

### *Aedes aegypti w*Mel*w*AlbA*w*AlbB crossing types

A series of individual male and female single crosses were set up involving the triple-infected and wild-type mosquitoes. After eggs from single crosses were obtained, the infection status was assessed by PCR and only the eggs from crosses involving PCR-confirmed triple infected individuals were hatched and used in assessing crossing types.

When triple-infected males were crossed to wild-type females, full CI was observed, with no eggs hatching. Low hatch rates were observed when triple-infected males and triple-infected females were crossed (21.3 ± 12.4%), in comparison to 76.5 ± 9.2% when wild-type males were crossed to triple-infected females, and 87.4 ± 4.1% for wild-type male and female control crosses (Table 3). Given incomplete maternal transmission, the low hatch rates in the triple-infected male and female within-line cross may result from either self-incompatibility, or incompatibility between triple infected males and progeny of triple infected females that do not inherit all three *Wolbachia* strains - or some combination of both. Less than half of eggs receive the full complement of *Wolbachia* strains (Table 2), and are therefore expected to experience incompatibility with sperm resulting from a triple-infected male.

**Table 3:** Percentage hatch rates (± SD) of eggs resulting from crosses between *Ae. aegypti* lines

		♂	
		*w*Mel*w*AlbA*w*AlbB	wt
♀	*w*Mel*w*AlbA*w*AlbB	21.3 ± 12.4	76.5 ± 9.2
	wt	0	87.4 ± 4.1

Each percentage shows the mean hatch rates from eggs resulting from ten separate single crosses, each containing a single female and a single male. For each cross > 50 eggs were assessed

The crossing types of the *Ae. aegypti w*AlbA*w*AlbB infection with the constituent *w*AlbA, *w*AlbB and wild type strains was assessed and showed full uni-directional CI with each (Table 4), and was self-compatible, as would be predicted. This lends support to the hypothesis that the presence of *w*Mel that was causing the unexpected crossing patterns in the triple-infected line.

**Table 4:** Crossing types of *Ae. aegypti* infected with either *w*AlbA-only, *w*AlbB-only, a superinfection of *w*AlbA and *w*AlbB, or uninfected (wt)

		♂			
		wt	*w*AlbA	*w*AlbB	*w*AlbA*w*AlbB
♀	wt	83.2	0	0	0
	*w*AlbA	81.7	77.4	0	0
	*w*AlbB	80.4	0	84.3	0
	*w*AlbA*w*AlbB	71.9	75.5	72.3	78.4

Numbers show mean hatch rates, with > 250 eggs counted for each cross

## Discussion

The *w*Mel-only infection in *Ae. albopictus* was previously reported to produce bidirectional CI with wild-type mosquitoes [7], but provided strong pathogen inhibition against the dengue [7] and chikungunya (8) viruses with minimal effects on host fitness [8]. We created a triple-infected line combining *w*Mel with the native strains *w*AlbA and *w*AlbB, with the aim of creating a *w*Mel-carrying line that produces unidirectional CI with wild populations, and is thus expected to have improved population invasion ability. While males of the triple infected *w*AlbA*w*AlbB*w*Mel line were able to successfully induce CI when crossed with wild-type females, the *w*AlbA*w*AlbB*w*Mel line showed self-incompatibility. The *w*AlbA*w*AlbB*w*Mel females could successfully rescue CI from *w*Mel-only males, but were incompatible with wild-type males, suggesting an inability to rescue CI from *w*AlbA and/or *w*AlbB. The observation of significantly reduced densities of *w*AlbA in *w*AlbA*w*AlbB*w*Mel ovaries compared to wild-type suggests that wAlbA in embryos reaches too low a density to be able to rescue the CI modification produced by the *w*AlbA strain in the males.

The self-incompatibility of the *w*Mel*w*AlbA*w*AlbB superinfection in *Ae. albopictus* means that it is unlikely to be a useful alternative for dengue control, and the *w*Mel-only *Ae. albopictus* line remains the best option for dengue transmission blocking in this species. Self-incompatibility will represent a potential natural barrier to new strains of *Wolbachia* successfully transferring into a host species that already contains one or more *Wolbachia* strains, if there are competitive interactions that reduce the density of one of the strains already present. The *w*Mel infection in the triple-infected *Ae. albopictus* line showed a wider tissue distribution compared to the native strains, which were strongly localised to the ovaries*.* This finding lends support to the hypothesis that co-evolutionary selective pressures favour the localisation of native *Wolbachia* infections to the germline over time.

Several native plus novel strain triple infections have previously been reported in *Ae. albopictus*. A triple-infection consisting of *w*AlbA, *w*AlbB and *w*Ri showed full uni-directional CI with wild-type mosquitoes and stable maternal transmission [14]. The difference in CI crossing patterns between the triple infections carrying either *w*Ri or *w*Mel may be a result of the high ovarian densities reached by *w*Mel, leading to higher levels of native strain exclusion. This suggests that an important consideration when generating further superinfected lines will be the selection of novel strains with minimal impacts on native strain density. In addition, *Ae. albopictus* triple infections have been generated carrying *w*AlbA, *w*AlbB and *w*Pip [18] and *w*AlbA, *w*AlbB and *w*MelPop [19]. In the case of the triple infection containing *w*MelPop, incomplete maternal transmission was observed when mosquitoes were fed a mouse-derived blood meal, with *w*MelPop suffering particularly low transmission fidelity. Moreover, eggs from *w*MelPop-containing triple-infected females displayed very low hatch rates in crosses to both triple-infected and wild-type (*w*AlbA*w*AlbB-carrying) males. Hatch rates were considerably higher (although still relatively low) when human blood was used. The authors suggest that the low hatch rates with mouse blood may be due to egg mortality resulting from elevated levels of nutrient depletion in the triple-infected embryos. An alternative explanation, supported by the present study, is that the low hatch rates are due to CI resulting from a degree of native strain exclusion in the ovaries, with the lower densities exacerbated on mouse blood. In *Drosophila simulans* the introduction of a third *Wolbachia* strain to an already naturally double-infected colony resulted in a stable triple infection, with high rates of maternal transmission and additive CI [20]. Naturally occurring triple infections have also been detected in the white fly, *Bemisia tabaci* [21], the adzuki bean beetle, *Callosobruchus chinensis* [22], and the parasitoid wasp, *Trichogramma ostriniae* [23].

We also transferred the triple infection into *Ae. aegypti*, where an unexpectedly high rate of *Wolbachia* strain transmission infidelity was observed. Given that the lines produced are all bidirectionally incompatible, the results presented here may indicate that an upper limit exists with certain strain combinations to the number of *Wolbachia* infections that can stably infect a line, particularly with novel hosts such as *Ae. aegypti*. This limit will likely be dependent on the densities of the infecting strains, and should be lower for multi-strain infections involving high density strains due to competitive interactions.

## Conclusions

This study presents results assessing the stability and population invasion potential for a *w*AlbA*w*AlbB*w*Mel triple *Wolbachia* infection in *Ae. albopictus* and *Ae. aegypti*. In *Ae. albopictus* the triple infection showed high rates of vertical transmission, but a self-incompatible CI phenotype, suggesting that the triple-infection would be unlikely to persist in wild populations. It is probable that the self-incompatible phenotype is a result of an observed displacement of the *w*AlbA strain in the ovaries of triple-infected females, which could result in diminished CI rescue. In *Ae. aegypti* the triple infection showed remarkably low maternal transmission fidelity. These results highlight the importance of inter-strain interactions in determining *Wolbachia* superinfection stability and therefore utility in vector-control.

## Methods

### Mosquito strains and rearing

The *Ae. albopictus* wild-type line was the Ascoli strain colonized from San Benedetto del Tronto, Italy in 2006 by G. Favia and colleagues. The uninfected line the UjuT strain generated by tetracycline treatment [17]. All mosquito colonies were maintained at 27 °C and 70% relative humidity with a 12-hour light/dark photocycle. Larvae were fed tropical fish pellets (Tetramin, Tetra, Melle, Germany) and adults were given access to a sucrose meal *ad libitum*. Blood meals were provided using a Hemotek artificial blood-feeding system (Hemotek, Blackburn, UK) using defribrinated sheep blood (TCS Biosciences, Botyl Claydon, UK). Eggs were collected by providing damp filter-paper (Grade 1 filter paper, Whatman plc, GE Healthcare, Coventry, UK) as an oviposition source and were desiccated for 5–10 days prior to hatching in water containing 1 g/l bovine liver powder (MP Biomedicals, Santa Ana, California, USA).

### Generation of *Wolbachia*-infected lines

The *w*AlbA*w*AlbB*w*Mel line was generated by transferring cytoplasm from *w*Mel-carrying *Ae. albopictus* embryos to wild-type (*w*AlbA*w*AlbB-carrying) *Ae. albopictus* embryos. The *Ae.aeg-w*AlbA*w*AlbB*w*Mel line was created by transferring cytoplasm from the triple infected (*w*AlbA*w*AlbB*w*Mel) *Ae. albopictus* line to wild-type (Malaysian) *Ae. aegypti*. Microinjections were performed using methods described previously [7]. Female G_0_ survivors were back-crossed to wild-type males, blood-fed and separated individually for oviposition. G_0_ females were analysed for *Wolbachia* infection by strain-specific PCR (see Additional file 1: Table S1 for sequences) and eggs from G_0_ females negative for the target transinfection were discarded. Eggs of positive females were hatched and G_1_’s were assessed for target transinfection G_0_-G_1_ germ-line transmission.

### CI and maternal inheritance

Rates of CI induction and rescue both with wild-type and *Wolbachia*-negative mosquitoes were assessed by crosses involving 20 males and 10 females in each of three separate cages. A blood meal was provided and females were individualised for oviposition. Eggs were collected on damp filter paper, which was subsequently desiccated for 5 days at 27 °C and 70% relative humidity. Eggs were counted and hatched in water containing 1 g/l bovine liver powder. Larvae were counted at the L2-L3 stage to provide hatch rates.

To assess rates of maternal inheritance, females from the *Wolbachia* transinfected lines were crossed to uninfected males in pools of 20 males and 10 females. A blood meal was provided and females were individualised for oviposition. Resulting eggs were hatched and DNA from a selection of the resulting pupae were analysed for specific *Wolbachia* strains by PCR.

### *Wolbachia* strain-specific PCR and density qPCR

For PCR analysis, genomic DNA was extracted from mosquitoes using the Livak method. For primer sequences see Additional file 1: Table S1. For measurements of *Wolbachia* density by qPCR, genomic DNA was extracted from mosquitoes using phenol/chloroform. Unless stated otherwise, mosquitoes used in density experiments were adults 5-days post-pupal eclosion. gDNA was diluted to 100 ng/μl using a NanoDrop spectrophotometer (Thermo Scientific, Waltham, Massachusetts, USA). A BioRad CFX-96 real-time PCR detection system was used (Bio Rad, Hercules, California, USA) with 2× SYBR-Green mastermix (Biotool, Houston, Texas, USA). Total *Wolbachia* density was analysed by absolute quantification against a dilution curve of a vector containing single copies of the homothorax (HTH) gene and *Wolbachia* surface protein (*wsp*).

To specifically quantify the *w*AlbA, *w*AlbB, and *w*Mel strains, the following primers were used: *w*AlbA - QAdir1 and QArev2; *w*AlbB - 183F and QBrev2; wMel - qMel-F and qMel-R. All were normalized against HTH copies. The following program was used to run the qPCRs: 95 **°**C for 5 min, 40× cycles of 95 **°**C for 15 s and 60 **°**C for 30 s, followed by a melt-curve analysis.

## Additional file

Additional file 1: **Table S1.** Primer sequences. (DOCX 16 kb)
