# Supplementary material for: A Wolbachia triple-strain infection generates self-incompatibility in Aedes albopictus and transmission instability in Aedes aegypti
Source: Parasit Vectors. 2018 May 11;11:295. doi: 10.1186/s13071-018-2870-0 (PMC5948879; doi:10.1186/s13071-018-2870-0)
Supplement: Supplementary file 1 — Table S1. Primer sequences. (DOCX 16 kb) [file 13071_2018_2870_MOESM1_ESM.docx]

**Additional file 1: Table S1 Primer sequences.**

| **Primer name** | **5’-3’ Sequence** |
| --- | --- |
| *w*AlbA-F | GTAGTATTTACCCCAGCAG |
| *w*AlbA-R | ATCTGCACCAGTAGTTTCG |
| *w*AlbB-F | GCAATACCTATGCCGTTTA |
| *w*AlbB-R | GACGAAGGGGATAGGTTAATATC |
| qMel-F | TATTGAGCCTTCCTCGTACC |
| qMel-R | TAGCATGCCGTTTTTCTGTA |
| qHTH-F [24] | TGGTCCTATATTGGCGAGCTA |
| qHTH-R [24] | TCGTTTTTGCAAGAAGGTCA |
| qWSP-F [1] | ATCTTTTATAGCTGGTGGTGGT |
| qWSP-R [1] | AAAGTCCCTCAACATCAACCC |
| QAdir1 [25] | GGGTTGATGTTGAAGGAG |
| QArev2 [25] | CACCAGCTTTTACTTGACC |
| 183F [24] | AAGGAACCGAAGTTCATG |
| QBrev2 [25] | AGTTGTGAGTAAAGTCCC |

**Additional references**

[24] Braig HR, Zhou W, Dobson SL, O’Neill SL. Cloning and characterization of a gene encoding the major surface protein of the bacterial endosymbiont Wolbachia pipientis. J Bacteriol. 1998;180:2373–8.

[25] Tortosa P, Courtiol A, Moutailler S, Failloux AB, Weill M. Chikungunya-Wolbachia interplay in Aedes albopictus. Insect Mol Biol. 2008;17:677–84.
